# The role of periodontitis in the link between alpha-tocopherol intake and cognitive performance: A mediation analysis in older adults

**DOI:** 10.3389/fnagi.2023.1129095

**Published:** 2023-03-09

**Authors:** Heming Zhang, Li Sun, Lin Zhang, Jiangjing Li, Yongfei Liu, Zhiyang Chen, Shuang Wang, Changjun Gao, Xude Sun

**Affiliations:** ^1^Department of Anesthesiology, The Second Affiliated Hospital of Air Force Medical University, Xi’an, China; ^2^Department of Anesthesiology, The 963 Hospital of the PLA Joint Logistics Support Force, Jiamusi, China; ^3^Department of Geriatric Cardiology, The Second Medical Center, Chinese PLA General Hospital, Beijing, China

**Keywords:** cognition, periodontitis, alpha-tocopherol, diet, National Health and Nutrition Examination Survey (NHANES)

## Abstract

**Background:**

Epidemiological evidence on alpha (α)-tocopherol intake and cognitive performance in older individuals is controversial and the effect of periodontitis in this chain is sparse and limited. The goal of this study was to characterize the association between α-tocopherol intake and cognitive performance and the mediating role of periodontitis in a nationally representative sample of older adults.

**Methods:**

Data from the National Health and Nutrition Examination Survey (NHANES), 2011–2014, were used. Multivariate logistic regression analysis was performed to explore the association of α-tocopherol intake, periodontal measures (mean attachment loss [AL] and mean probing depth [PD]), and clinical periodontitis defined by the European Workshop in Periodontology with poor cognitive performance evaluated by Consortium to Establish a Registry for Alzheimer’s disease (CERAD); the animal fluency test (AFT); and the Digit Symbol Substitution test (DSST) and the correlation between α-tocopherol intake and clinical periodontitis. Multiple linear regression analysis was used to explore the relationship between α-tocopherol intake and periodontal measures. Mediation analysis was used to test the effects of periodontal measures on the association between α-tocopherol intake and cognitive measures.

**Results:**

A total of 1,749 older participants (≥60 years of age) with complete periodontal diagnosis, dietary retrospective survey, and cognitive tests were included. In the fully adjusted model, the odds ratio (OR) with 95% confidence interval (CI) of CERAD score, AFT score and DSST score were 0.214 (0.137–0.327), 0.378 (0.241–0.585) and 0.298 (0.169–0.512) for the highest versus lowest tertile of α-tocopherol intake, respectively. And participants with clinical periodontitis were more likely to exhibit lower DSST score (OR = 1.689; 95 CI%: 1.018–2.771) than those without periodontitis. Mean AL (OR = 1.296; 95 CI%: 1.102–1.524) and PD (OR = 1.667; 95 CI%: 1.18–2.363) were negatively correlated with DSST, and were estimated to mediate 9.1 and 8.2% of the total association between α-tocopherol intake and cognitive performance, respectively.

**Conclusion:**

Finding of the present study suggested that participants with low α-tocopherol intake were at higher risk for developing cognitive decline. Moreover, periodontitis mediated the association between α-tocopherol intake and cognitive performance.

## 1. Introduction

Populations are aging rapidly worldwide, and the number of older individuals ≥60 years of age is expected to increase from 962 million in 2017 to 2.1 billion by 2050 (Li et al., 2018). Aging-associated cognitive decline, characterized by impairment of intellectual abilities, such as memory, executive function, sustained attention, and processing speed, can be a major health challenge for the older individuals. To date, understanding cognitive decline and its reason has been greatly accomplished, such as inflammation, oxidative stress and extracellular plaques. And nutrients perhaps play a critical role in these mechanisms of cognitive decline. In addition, there are many risk factors for cognitive decline has been accomplished including periodontitis.

Periodontitis is a common chronic oral inflammatory disease (Sung et al., 2019), and is characterized by a polymicrobial dysbiotic infection of the periodontium (Hajishengallis, 2015). Evidence suggests that *Porphyromonas gingivalis*, a keystone pathogen of periodontitis, has been identified as a significant etiological factor in the development of amyloid-beta peptide (Aβ) plaques and Alzheimer’s disease (AD) (Dominy et al., 2019). In addition, clinical studies have shown that patients with periodontitis tend to exhibit poorer cognitive performance (Shin et al., 2016).

Previous studies have examined the effects of vitamin E supplementation on cognitive performance, and the results are complex and controversial (Dysken et al., 2014; Kryscio et al., 2017; Rutjes et al., 2018). Several studies have shown that alpha (α)-tocopherol, a form of vitamin E, can be used to treat AD through its anti-inflammatory and antioxidant effects, whereas others have reported inconsistent results indicating that vitamin E including α-tocopherol has no benefit for those with AD (Lloret et al., 2019). Nevertheless, previous meta-analysis has shown that lower peripheral α-tocopherol levels is associated with AD and mild cognitive impairment (Ashley et al., 2021). At the same time, several studies investigating the causal relationship between α-tocopherol intake and periodontitis have reported that dietary α-tocopherol intake exerts a positive effect on chronic periodontitis (Dodington et al., 2015; Zong et al., 2015). Thus, individuals with low α-tocopherol intake may experience periodontitis, which in turn, can adversely influence cognitive performance.

The above evidence may suggest that periodontitis plays a role in the causal chain between α-tocopherol intake and cognitive performance. However, it remains unclear whether periodontitis plays a mediating role in the relationship between α-tocopherol intake and cognitive performance. In the present study, we aimed to explore possible correlations between α-tocopherol intake and cognitive performance among older participants from the National Health and Nutrition Examination Survey (NHANES) 2011–2014 cohort, and further investigated whether this relationship was mediated by periodontitis.

## 2. Materials and methods

### 2.1. Data sources

Data were collected from two cycles of the NHANES dataset (2011–2012 and 2013–2014), a cross-sectional survey conducted by the Centers for Disease Control and Prevention (Atlanta, GA, USA). The Research Ethics Review Board of the National Center for Health Statistics (NCHS) approved the survey protocol. All participants provided written informed consent before participating in the study.

### 2.2. Study population

**FIGURE 1:**
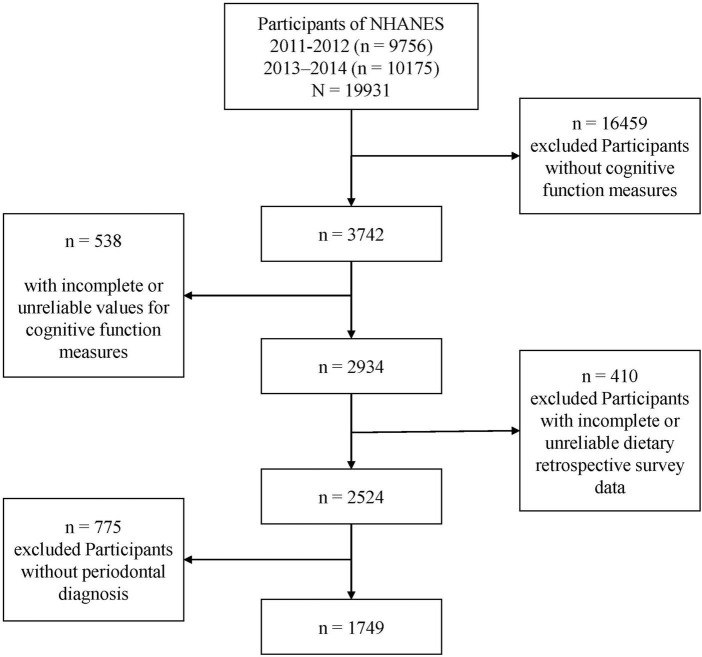
Flow chart for the selection of included sample.

In total, 19,931 participants in NHANES during 2011–2014 were included. Participants with missing or unreliable data on cognitive function measures, dietary retrospective survey, and periodontal diagnosis were excluded from our analysis. Information from 3,742 older participants with available cognitive performance data was extracted from the NHANES database for 2011–2014. Among these, participants with incomplete or unreliable data for cognitive performance measures (*n* = 538) and/or retrospective dietary surveys (*n* = 410) were excluded. Additionally, individuals with no periodontal diagnosis (*n* = 775) were also excluded. Ultimately, data from 1,749 participants were included in the present study (Figure 1).

### 2.3. Cognitive test battery

Cognitive performance was assessed using a series of tests, including: Consortium to Establish a Registry for Alzheimer’s disease (CERAD); the animal fluency test (AFT); and the Digit Symbol Substitution test (DSST).

The CERAD test, a standardized and validated measures for the assessment of AD, consisted of three consecutive learning trials and one delayed recall trial, which were designed to evaluate episodic memory (Bailey et al., 2020). After learning, the participants were required to recall as many words as possible. The CERAD score represents the total score of the four tests. The AFT assesses verbal fluency and semantic-based memory function (Bailey et al., 2020). It has been shown to discriminate between persons with normal cognitive functioning compared with those with Alzheimer’s disease (Clark et al., 2009). Participants who passed the sample practice pretest were asked to name as many animals as possible in 1 min. The DSST was used to evaluate processing speed, sustained attention, and working memory of the participants. Participants were given 2 min to copy corresponding symbols in 133 boxes that adjoined the numbers (Brody et al., 2019). According to the cut-off points (Table 1), participants were divided into low and normal cognition performance groups.

**TABLE 1:** The cognition performance cut-off points of test score, adjusted according to age.

	CERAD score	AFT score	DSST score
60–69 years 70–79 years ≥80 years	23 20 17	15 12 11	39 35 30

CERAD, Consortium to Establish a Registry for Alzheimer’s disease; AFT, animal fluency test; DSST, Digit Symbol Substitution test.

### 2.4. Dietary intake assessment

α-tocopherol intake data were obtained from two 24 h dietary recall interviews. The first interview was conducted simultaneously with the Mobile Examination Center (MEC) examination, and the second was conducted 3–10 days later. In this analysis, the total dietary intake of α-tocopherol was calculated by averaging the data from the two dietary recalls. Single dietary data points were excluded from the study. The sum of two parameters, “vitamin E as alpha-tocopherol” and “added alpha-tocopherol” in the NHANES 2011–2014 was defined as the α-tocopherol intake. Dietary α-tocopherol intake was categorized into quartiles (Q1, Q2, Q3, and Q4).

### 2.5. Periodontal assessment

Periodontal assessment, a part of MEC examination, was conducted using an oral health program according to the NHANES Oral Health Examiners Manual. Attachment loss (AL) and pocket depth (PD) were measured to the nearest distance from the bottom of the pocket to the cementum enamel junction or the marginal gingiva, respectively. And they were recorded at 6 sites per tooth (mesio-buccal, mid-buccal, disto-buccal, mesio-lingual, mid-lingual, and disto-lingual). Mean AL and mean PD were calculated. According to the European Workshop in Periodontology, clinical periodontitis was defined as the presence of proximal AL ≥ 5 mm in ≥30% of teeth present (Kongstad et al., 2017).

### 2.6. Covariates

Some potential confounding factors that were investigated are summarized in Table 2. Smoking was classified as follows: never smoker (never smoked or smoked < 100 cigarettes in life); former smoker (smoked ≥ 100 cigarettes in life and quit smoking); or current smoker (smoked ≥ 100 cigarettes in life and currently smoking). Alcohol consumption was defined as the consumption of at least 12 alcoholic drinks per year. Depression was assessed using the Patient Health Questionnaire-9. The responses “not at all,” “several days,” “more than half the days,” and “nearly every day” were assigned a score ranging from 0 to 3. The total score is based on the sum of the points for each item, ranging from 0 to 27. Diabetes, hypertension, and stroke were defined as having been diagnosed as such by a physician.

**TABLE 2:** The classifications of covariates.

Covariates	Classifications
Gender	Male; female
Age (year)	60–69; 70–79; ≥80
Race	MA; hispanic; NHW; NHB; NHA; others
Educational level	Below high school; high school; above high school
Marital status	Never married; married; divorced; widowed; other
PIR	<1; and ≥1
BMI	<18.5 kg/m^2^; 18.5 to <25 kg/m^2^; 25 to <30 kg/m^2^; ≥30 kg/m^2^
Smoking	Never smoker; former smoker; current smoker
Alcoholic drinking	No; yes
Depression	Patient Health Questionnaire-9 score
Hypertension	No; yes
Diabetes	No; yes
Stroke	No; yes
Work activity	Vigorous; moderate; other
Recreational activity	Vigorous; moderate; other

MA, Mexican American; NHW, non-hispanic white; NHB, non-hispanic black; NHA, non-Hispanic Asian; PIR, poverty-income ratio; BMI, body mass index.

### 2.7. Statistical analysis

All statistical analyses were performed using R version 4.2.1 (R Foundation for Statistical Computing, Vienna, Austria). Based on the analytical guidelines of the National Health and NHANES, new sample weights were constructed. Missing data were displayed using the R package “VIM” and complemented by multiple imputation. And the R package “survey” was used to analyze statistical differences in different groups and establish regression model. Non-normally distributed data are expressed as median and interquartile range (IQR) and compared using Mood’s test. Count data are expressed as number of cases and composition ratio (n [%]), and comparisons were performed using the χ2 or Fisher’s exact tests.

Multivariate logistic regression analysis was performed to explore whether α-tocopherol intake, mean AL, mean PD, and clinical periodontitis were associated with low cognitive performance according to CERAD, AFT, and DSST, and to further assess the relationship between α-tocopherol intake and clinical periodontitis. Q1 was used as the reference group for higher α-tocopherol intake. The results are expressed odds ratio (OR) and corresponding 95% confidence interval (CI) which calculated by R package “stats.” Multiple linear regression analysis was used to measure the association of α-tocopherol intake with two periodontal measures (mean AL and mean PD), with the results reported as β and corresponding 95% CI. *F*-test was used to check the assumptions of linear regression analysis. In the analysis, model 1 was not adjusted for confounders; model 2 was adjusted for age, sex, and body mass index (BMI); and model 3 was adjusted for age, sex, race, educational level, marital status, poverty-income ratio (PIR), BMI, smoking, drinking, depression, diabetes, hypertension, stroke, work activity, and recreational activities.

**FIGURE 2:**
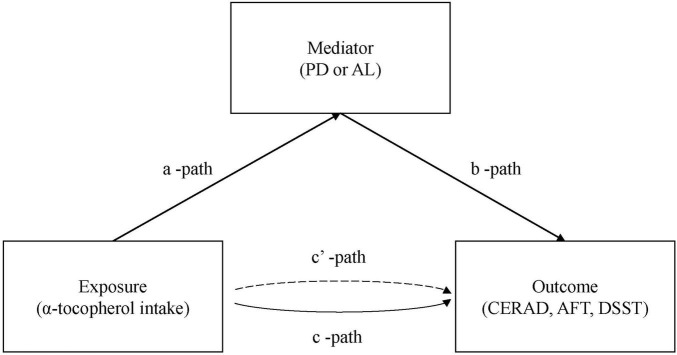
Path diagram of the mediation analysis models.

The mediating effect of mean PD and mean AL in the association of α-tocopherol intake with all three cognitive measures were determined using the R package “mediation.” The regression models for the mediation analysis were adjusted for all covariates in this study. Figure 2 provides a path model that indicates the mediation effect of periodontal measures. Differences with a two-sided *P* < 0.05 were considered to be statistically significant.

## 3. Results

Of 2,934 participants with complete and reliable cognitive performance test data, 1,749 with complete and reliable dietary retrospective survey data and periodontal data were included in the present study (Figure 1). Missing data for the selected variables were <10% of the total sample (Supplementary Figure 1). In this study, missing data were complemented using multiple imputations.

The baseline characteristics of the study sample (*n* = 1749) divided according to the level of α-tocopherol intake are summarized in Table 3. Significant differences were observed among the participants in different groups according to gender (*p* < 0.001), education (*p* < 0.001), marital status (*p* = 0.001), PIR (*p* < 0.001), smoking (*p* = 0.035), alcohol consumption (*p* < 0.001), hypertension (*p* = 0.028), recreational activity (*p* = 0.003), periodontal assessment (*p* < 0.001), CERAD (*p* < 0.001), AFT (*p* < 0.001), and DSST (*p* < 0.001), but not according to age, race, BMI, depression score, diabetes, stroke, and work activity.

**TABLE 3:** Characteristics of the included participants (*n* = 1749).

Characteristics	Total sample	Quartile of α-tocopherol intake (mg/day)				*P*-value
		**Q1 (≤4.925)**	**Q2 (4.925 to ≤7.25)**	**Q3 (7.25 to ≤10.435)**	**Q4 (>10.435)**	
Number of participants, *n*	1749	438	437	437	437	
Gender, *n* (%)						<0.001
Male	857 (49)	178 (40.6)	192 (43.9)	253 (57.9)	234 (53.5)	
Female	892 (51)	260 (59.4)	245 (56.1)	184 (42.1)	203 (46.5)	
Age (years), *n* (%)						0.284
60–69	1049 (59.98)	254 (58.0)	258 (59.0)	255 (58.4)	282 (64.5)	
70–79	469 (26.82)	123 (28.1)	123 (28.1)	120 (27.5)	103 (23.6)	
≥80	231 (13.21)	61 (13.9)	56 (12.8)	62 (14.2)	52 (11.9)	
Race, *n* (%)						0.071
MA	161 (9.21)	36 (8.2)	43 (9.8)	45 (10.3)	37 (8.5)	
Hispanic	175 (10.01)	55 (12.6)	50 (11.4)	46 (10.5)	24 (5.5)	
NHW	859 (49.11)	185 (42.2)	199 (45.5)	217 (49.7)	258 (59.0)	
NHB	398 (22.76)	120 (27.4)	105 (24.0)	97 (22.2)	76 (17.4)	
NHA	134 (7.66)	35 (8.0)	37 (8.5)	25 (5.7)	37 (8.5)	
Other	22 (1.26)	7 (1.6)	3 (0.7)	7 (1.6)	5 (1.1)	
Education, *n* (%)						<0.001
Below high school	358 (20.47)	137 (31.3)	98 (22.4)	71 (16.2)	52 (11.9)	
High school	407 (23.27)	114 (26.0)	107 (24.5)	98 (22.4)	89 (20.4)	
Above high school	984 (56.26)	187 (42.7)	232 (53.1)	268 (61.3)	296 (67.7)	
Marital status, *n* (%)						0.001
Never married	106 (6.06)	29 (6.6)	23 (5.3)	23 (5.3)	31 (7.1)	
Married	1011 (57.8)	208 (47.5)	258 (59.0)	265 (60.6)	280 (64.1)	
Divorced	244 (13.95)	69 (15.8)	59 (13.5)	61 (14.0)	55 (12.6)	
Widowed	282 (16.12)	98 (22.4)	69 (15.8)	57 (13.0)	58 (13.3)	
Other	106 (6.06)	34 (7.8)	28 (6.4)	31 (7.1)	13 (3.0)	
Poverty income ratio, *n* (%)						<0.001
<1	224 (12.81)	88 (20.1)	57 (13.0)	48 (11.0)	35 (8.0)	
≥1	1525 (87.19)	350 (79.9)	380 (87.0)	389 (89.0)	402 (92.0)	
BMI, (kg/m^2^), *n* (%)						0.228
<18.5	23 (1.32)	8 (1.8)	8 (1.8)	2 (0.5)	6 (1.4)	
18.5 to <25	438 (25.04)	100 (22.8)	104 (23.8)	117 (26.8)	115 (26.3)	
25 to <30	624 (35.68)	145 (33.1)	165 (37.8)	151 (34.6)	164 (37.5)	
≥30	664 (37.96)	185 (42.2)	160 (36.6)	167 (38.2)	152 (34.8)	
Smoking, *n* (%)						0.035
Never	914 (52.26)	242 (55.3)	246 (56.3)	205 (46.9)	221 (50.6)	
Former	652 (37.28)	135 (30.8)	158 (36.2)	186 (42.6)	173 (39.6)	
Current	183 (10.46)	61 (13.9)	33 (7.6)	46 (10.5)	43 (9.8)	
Alcohol consumption, *n* (%)	1223 (69.93)	267 (61.0)	289 (66.1)	328 (75.1)	338 (77.3)	<0.001
Depression score, median (IQR)	1 (0–4)	1 (2–5)	1 (0–4)	1 (0–3)	1 (0–4)	0.114
Hypertension, *n* (%)	1031 (58.95)	282 (64.4)	263 (60.2)	244 (55.8)	244 (55.8)	0.028
Diabetes, *n* (%)	379 (21.67)	112 (25.6)	99 (22.7)	90 (20.6)	78 (17.8)	0.088
Stroke, *n* (%)	86 (4.92)	26 (5.9)	19 (4.3)	19 (4.3)	22 (5.0)	0.882
Work activity, *n* (%)						0.53
Vigorous	213 (12.18)	47 (10.7)	52 (11.9)	59 (13.5)	55 (12.6)	
Moderate	371 (21.21)	75 (17.1)	83 (19.0)	100 (22.9)	112 (25.6)	
Other	1165 (66.61)	316 (72.1)	302 (69.1)	278 (63.6)	270 (61.8)	
Recreational activity, *n* (%)						0.003
Vigorous	203 (11.61)	28 (6.4)	49 (11.2)	51 (11.7)	75 (17.2)	
Moderate	616 (35.22)	128 (29.2)	168 (38.4)	162 (37.1)	158 (36.2)	
Other	930 (53.17)	282 (64.4)	220 (50.3)	224 (51.3)	204 (46.7)	
Periodontal assessment, *n* (%)						<0.001
Periodontitis	259 (14.81)	80 (18.26)	63 (14.42)	60 (13.73)	56 (12.81)	
Non-periodontitis	1493 (85.19)	358 (81.74)	374 (85.58)	377 (86.27)	381 (87.19)	
CERAD, *n* (%)						<0.001
Normal cognitive performance	1378 (78.79)	299 (68.26)	352 (80.55)	353 (80.78)	374 (85.58)	
Low cognitive performance	371 (21.21)	139 (31.74)	85 (19.45)	84 (19.22)	63 (14.42)	
AFT, *n* (%)						<0.001
Normal cognitive performance	1335 (76.33)	287 (65.53)	321 (73.46)	353 (80.78)	374 (85.58)	
Low cognitive performance	414 (23.67)	151 (34.47)	116 (26.54)	84 (19.22)	63 (14.42)	
DSST, *n* (%)						<0.001
Normal cognitive performance	1354 (77.42)	280 (63.93)	331 (75.74)	364 (83.3)	379 (86.73)	
Low cognitive performance	395 (22.58)	158 (36.07)	106 (24.26)	73 (16.7)	58 (13.27)	

MA, Mexican American; NHW, non-hispanic white; NHB, non-hispanic black; NHA, non-Hispanic Asian; PIR, poverty-income ratio; BMI, body mass index; IQR, interquartile range; CERAD, Consortium to Establish a Registry for Alzheimer’s disease; AFT, animal fluency test; DSST, Digit Symbol Substitution test.

Data regarding the association of α-tocopherol intake with low cognitive performance are summarized in Table 4. Compared with the lowest α-tocopherol intake group, the higher quartile of α-tocopherol intake group was positively correlated with CERAD, AFT, and DSST in all three models, except for the Q2 α-tocopherol intake group in the AFT in model 3. The association of mean AL, mean PD, and clinical periodontitis with low cognitive performance are shown in Table 5. Mean AL, PD, and clinical periodontitis were negatively associated with all three cognitive assessments in models 1 and 2. When all covariates were adjusted for in this study, the associations of mean AL (OR = 1.296; 95 CI%: 1.102–1.524), mean PD (OR = 1.667; 95 CI%: 1.18–2.363), and clinical periodontitis (OR = 1.689; 95 CI%: 1.018–2.771) with DSST were attenuated but remained significant, whereas the associations with CERAD scores were no longer significant. In terms of AFT, clinical periodontitis (OR = 1.694; 95 CI%: 1.081–2.624) was still significantly negatively correlated with AFT, whereas no significant association of mean AL and mean PD with AFT was observed in model 3.

**TABLE 4:** Associations of quartile of α-tocopherol intake with low cognitive performance.

	Model 1		Model 2		Model 3	
	**OR (95% CI)**	***P*** **-value**	**OR (95% CI)**	***P*** **-value**	**OR (95% CI)**	***P*** **-value**
**CERAD**						
**α-tocopherol intake(mg/day)**						
Q1 (≤4.925)	Referent	Referent	Referent	Referent	Referent	Referent
Q2 (4.925 to ≤7.25)	0.39 (0.273–0.554)	<0.001	0.349 (0.242–0.499)	<0.001	0.395 (0.268–0.579)	<0.001
Q3 (7.25 to ≤10.435)	0.427 (0.303–0.597)	<0.001	0.371 (0.261–0.524)	<0.001	0.471 (0.323–0.682)	<0.001
Q4 (>10.435)	0.192 (0.127–0.284)	<0.001	0.164 (0.107–0.245)	<0.001	0.214 (0.137–0.327)	<0.001
**AFT**						
**α-tocopherol intake (mg/day)**						
Q1 (≤4.925)	Referent	Referent	Referent	Referent	Referent	Referent
Q2 (4.925 to ≤7.25)	0.679 (0.48–0.959)	0.028	0.669 (0.471–0.948)	0.024	0.861 (0.584–1.268)	0.448
Q3 (7.25 to ≤10.435)	0.402 (0.275–0.582)	<0.001	0.39 (0.265–0.567)	<0.001	0.531 (0.348–0.803)	0.003
Q4 (>10.435)	0.272 (0.18–0.404)	<0.001	0.26 (0.172–0.389)	<0.001	0.378 (0.241–0.585)	<0.001
**DSST**						
**α-tocopherol intake (mg/day)**						
Q1 (≤4.925)	Referent	Referent	Referent	Referent	Referent	Referent
Q2 (4.925 to ≤7.25)	0.409 (0.274–0.602)	<0.001	0.429 (0.291–0.628)	<0.001	0.527 (0.322–0.854)	0.01
Q3 (7.25 to ≤10.435)	0.251 (0.162–0.383)	<0.001	0.275 (0.179–0.414)	<0.001	0.342 (0.201–0.572)	<0.001
Q4 (>10.435)	0.172 (0.105–0.271)	<0.001	0.183 (0.113–0.286)	<0.001	0.298 (0.169–0.512)	<0.001

CERAD, Consortium to Establish a Registry for Alzheimer’s disease; AFT, animal fluency test; DSST, Digit Symbol Substitution test; OR, odds ratio; CI, confidence interval.

**TABLE 5:** Associations of mean AL, mean PD, and periodontitis with low cognitive performance.

	Model 1		Model 2		Model 3	
	**OR (95% CI)**	***P*** **-value**	**OR (95% CI)**	***P*** **-value**	**OR (95% CI)**	***P*** **-value**
**CERAD**						
Mean AL	1.3 (1.169–1.443)	<0.001	1.246 (1.117–1.388)	<0.001	1.019 (0.891–1.161)	0.779
Mean PD	1.749 (1.382–2.211)	<0.001	1.652 (1.296–2.1)	<0.001	1.24 (0.944–1.624)	0.12
Periodontitis	2.017 (1.359–2.942)	<0.001	1.794 (1.199–2.638)	0.004	1.057 (0.671–1.636)	0.806
**AFT**						
Mean AL	1.305 (1.171–1.451)	<0.001	1.333 (1.193–1.488)	<0.001	1.055 (0.918–1.209)	0.447
Mean PD	1.395 (1.086–1.779)	0.008	1.407 (1.09–1.805)	0.008	0.928 (0.692–1.238)	0.615
Periodontitis	2.746 (1.872–3.975)	<0.001	2.861 (1.933–4.181)	<0.001	1.694 (1.081–2.624)	0.02
**DSST**						
Mean AL	1.738 (1.552–1.949)	<0.001	1.744 (1.549–1.967)	<0.001	1.296 (1.102–1.524)	0.002
Mean PD	2.821 (2.177–3.67)	<0.001	2.818 (2.154–3.704)	<0.001	1.667 (1.18–2.363)	0.004
Periodontitis	4.181 (2.836–6.096)	<0.001	3.979 (2.665–5.881)	<0.001	1.689 (1.018–2.771)	0.04

CERAD, Consortium to Establish a Registry for Alzheimer’s disease; AFT, animal fluency test; DSST, Digit Symbol Substitution test; OR, odds ratio; CI, confidence interval; AL, attachment loss; PD, pocket depth.

The associations of α-tocopherol intake with mean AL and PD are summarized in Table 6. Compared with the lowest α-tocopherol intake group, the higher quartile of α-tocopherol intake group exhibited a significant negative correlation with the mean AL and PD in all three models. And the results of *F*-test were statistically different in all models (*P* < 0.05). In models 1 and 2, the higher α-tocopherol intake quartile was significantly positively associated with clinical periodontitis compared to the lowest α-tocopherol intake quartile (Table 7). After all covariates adjustment in model 3, the associations between α-tocopherol intake and clinical periodontitis were still significant in the third (OR = 0.582; 95 CI%: 0.348–0.967) and fourth (OR = 0.481; 95 CI%: 0.277–0.823) quartiles of α-tocopherol intake, whereas no significant association was observed in the second quartile of α-tocopherol intake.

**TABLE 6:** β (p) of mean PD and mean AL according to the quartile of α-tocopherol intake.

	Model 1		Model 2		Model 3	
	**β (95% CI)**	***P*** **-value**	**β (95% CI)**	***P*** **-value**	**β (95% CI)**	***P*** **-value**
**Mean AL**						
**α-tocopherol intake (mg/day)**						
Q1 (≤4.925)	Referent	Referent	Referent	Referent	Referent	Referent
Q2 (4.925 to ≤7.25)	−0.37 (−0.516 to −0.224)	<0.001	−0.417 (−0.559 to −0.275)	<0.001	−0.265 (−0.394 to −0.136)	<0.001
Q3 (7.25 to ≤10.435)	−0.332 (−0.475 to −0.189)	<0.001	−0.416 (−0.556 to −0.276)	<0.001	−0.228 (−0.356 to −0.1)	<0.001
Q4 (>10.435)	−0.403 (−0.543 to −0.262)	<0.001	−0.474 (−0.611 to −0.336)	<0.001	−0.234 (−0.361 to −0.106)	<0.001
**Mean PD**						
**α-tocopherol intake (mg/day)**						
Q1 (≤4.925)	Referent	Referent	Referent	Referent	Referent	Referent
Q2 (4.925 to ≤7.25)	−0.16 (−0.23 to −0.091)	<0.001	−0.171 (−0.24 to −0.103)	<0.001	−0.121 (−0.186 to −0.055)	<0.001
Q3 (7.25 to ≤10.435)	−0.11 (−0.178 to −0.042)	0.002	−0.142 (−0.209 to −0.074)	<0.001	−0.084 (−0.149 to −0.019)	0.012
Q4 (>10.435)	−0.165 (−0.232 to −0.098)	<0.001	−0.191 (−0.258 to −0.125)	<0.001	−0.11 (−0.175 to −0.046)	<0.001

AL, attachment loss; PD, pocket depth; CI, confidence interval.

**TABLE 7:** Associations of quartile of α-tocopherol intake with periodontitis defined by EWP definition.

	Model 1		Model 2		Model 3	
	**OR (95% CI)**	***P*** **-value**	**OR (95% CI)**	***P*** **-value**	**OR (95% CI)**	***P*** **-value**
**α-tocopherol intake (mg/day)**						
Q1 (≤4.925)	Referent	Referent	Referent	Referent	Referent	Referent
Q2 (4.925 to ≤7.25)	0.544 (0.346–0.845)	0.007	0.476 (0.299–0.749)	0.002	0.7 (0.419–1.16)	0.168
Q3 (7.25 to ≤10.435)	0.492 (0.313–0.765)	0.002	0.4 (0.251–0.63)	<0.001	0.582 (0.348–0.967)	0.038
Q4 (>10.435)	0.338 (0.207–0.541)	<0.001	0.269 (0.162–0.437)	<0.001	0.481 (0.277–0.823)	0.008

EWP, European Workshop in Periodontology; OR, odds ratio; CI, confidence interval.

The mediating effect of mean AL on the association between α-tocopherol intake and cognitive performance tests is shown in Table 8. After adjusting for all confounding factors, the mediation effect of mean AL was found in the DSST (β = −0.005; 95% CI: −0.0126 to −0.001). However, no significant mediation effect of mean AL on the association of α-tocopherol intake with CERAD and AFT was observed. Similar to the mean AL, the mediation effect of mean PD was found in DSST (β = −0.0045; 95% CI: −0.0104 to −0.0008) (Table 9). However, no significant mediation effect of mean PD on the association of α-tocopherol intake with CERAD and AFT was observed. It was estimated that 9.1 and 8.2% of the total association between α-tocopherol intake and DSST was mediated by the mean AL and PD, respectively.

**TABLE 8:** The mediating proportion of mean AL on the association between α-tocopherol intake and low cognitive performance.

	Direct effect		Indirect effect		Total effect		Proporation mediated (%)
	**β (95% CI)**	***P*** **-value**	**β (95% CI)**	***P*** **-value**	**β (95% CI)**	***P*** **-value**	
CERAD	–0.1304 (–0.2029 to –0.0602)	<0.01	0.001 (–0.0047 to 0.0063)	0.74	–0.1294 (–0.2039 to –0.0594)	<0.01	–
AFT	–0.0097 (–0.07 to 0.0519)	0.84	–0.0008 (–0.0096 to 0.0061)	0.92	–0.0105 (–0.0679 to 0.0514)	0.8	–
DSST	–0.0463 (–0.0867 to –0.0097)	0.04	–0.005 (–0.0126 to –0.001)	<0.01	–0.0512 (–0.0919 to –0.017)	0.04	9.1

CERAD, Consortium to Establish a Registry for Alzheimer’s disease; AFT, animal fluency test; DSST, Digit Symbol Substitution test; CI, confidence interval.

**TABLE 9:** The mediating proportion of mean PD on the association between α-tocopherol intake and low cognitive performance.

	Direct effect		Indirect effect		Total effect		Proporation mediated (%)
	**β (95% CI)**	***P*** **-value**	**β (95% CI)**	***P*** **-value**	**β (95% CI)**	***P*** **-value**	
CERAD	–0.1343 (–0.2032 to –0.0641)	<0.01	–0.0025 (–0.0099 to 0.0038)	0.32	–0.1368 (–0.209 to –0.0647)	<0.01	–
AFT	–0.02 (–0.0855 to 0.0517)	0.5	0.0019 (–0.0039 to 0.0096)	0.54	–0.0181 (–0.0831 to 0.0535)	0.52	–
DSST	–0.0481 (–0.0939 to –0.0078)	0.02	–0.0045 (–0.0104 to –0.0008)	<0.01	–0.0526 (–0.0983 to –0.011)	0.02	8.2

CERAD, Consortium to Establish a Registry for Alzheimer’s disease; AFT, animal fluency test; DSST, Digit Symbol Substitution test; CI, confidence interval.

## 4. Discussion

The present study investigated the relationship between α-tocopherol intake and cognitive performance and the mediating effect of periodontitis and found that α-tocopherol intake deficient was inversely correlated with cognitive performance among older adults. In addition, periodontitis significantly mediated the association between α-tocopherol intake and cognitive performance in terms of processing speed, sustained attention, and working memory.

Our results regarding the relationship between α-tocopherol levels and cognitive performance are consistent with those reported in previous studies. A prospective study involving 960 residents, ages 58–99 years, who were followed up for a mean of 4.7 years, reported that foods containing α-tocopherol may slow cognitive decline with aging (Morris et al., 2018). In addition, an analysis, including 341 AD patients, also indicated a modest delay in the disease progression of AD among those with greater consumption of α-tocopherol (Sano et al., 1997). Furthermore, evidence suggests that low serum α-tocopherol levels are significantly correlated with memory and mixed impairments (Dunn et al., 2007). However, study with inconsistent results indicated that α-tocopherol supplementation couldn’t protect aging-related cognitive impairment (Alghadir et al., 2021). That may be due to the complexity of α-tocopherol bioavailability and it can be influenced by factors including age, obesity and genetic polymorphisms. In this study, we chose the dietary as the main approach to calculate the amount of daily α-tocopherol intake, and low-level daily α-tocopherol intake was significantly correlated with cognitive decline.

The effect of α-tocopherol intake on cognitive performance may be partially mediated by periodontitis. Existing observational studies have indicated that serum α-tocopherol has a non-linear inverse association with periodontitis in adults (Zong et al., 2015). In a cohort study, 4,559 participants were followed up for 15 years and periodontitis was found to be associated with a reduced risk for cognitive decline (Demmer et al., 2020). In this study, we first investigated the relationship between α-tocopherol intake, periodontitis, and cognitive performance through regression analysis, and then build models included different covariates to control variables that might affect cognitive performance. The results revealed that α-tocopherol intake was significantly associated with periodontitis, and so was the relationship between periodontitis and partial aspects of cognitive performance. Therefore, adequate α-tocopherol intake in older individuals with periodontitis may help to maintain better cognitive performance. Even a small reduction in the risk for cognitive decline could have significant health benefits for the public because of the large older individual base.

There are several possible mechanisms that explain the mediating effect of periodontitis on α-tocopherol intake and cognitive performance in older adults. First, α-tocopherol has been indicated to decrease levels of interleukin (IL)-1β and IL-6 produced by gingival fibroblasts. It can also increase various human β-defensins to defend against the adverse effects of LPS from periodontal bacteria (Derradjia et al., 2016). In addition, α-tocopherol regulates the production of oxygen free radicals by neutrophils following activation by Fcγ receptor and toll-like receptor ligands (Chapple et al., 2013). The antioxidant effect of α-tocopherol could resist oxidative stress damage from periodontitis to prevent cognitive performance (La Fata et al., 2014).

The present study had several strengths. First, the sample size was sufficiently large, and quality control of the NHANES database is excellent. Second, we adjusted for important confounding factors to precisely estimate the mediating effect of periodontitis on α-tocopherol intake and cognitive performance.

However, some limitations of the present investigation should not be ignored. First, it was difficult to determine causality due its cross-sectional design. Second, cognitive assessments in this study did not fully reflect cognitive performance because of the complexity of cognition. Third, α-tocopherol is only one form of vitamin E, and its effect could not fully represent the entire function of vitamin E due to limitations of data housed in the NHANES database.

## 5. Conclusion

The results of this study suggest that appropriate dietary α-tocopherol intake may reduce periodontitis and, thus, maintain better cognitive performance in individuals ≥60 years of age in the United States. It is noteworthy that dietary α-tocopherol intake is an important and effective means to protect cognitive performance. Appropriate dietary α-tocopherol intake should be provided, even if there is a controversial cognitive protective effect of α-tocopherol supplementation strategy on mild cognitive impairment and AD. Given the rational findings and several limitations in this study, the results should be further confirmed in the large prospective cohort study.

## Data availability statement

Publicly available datasets were analyzed in this study. This data can be found here: https://www.cdc.gov/nchs/index.htm.

## Ethics statement

The studies involving human participants were reviewed and approved by the Research Ethics Review Board of the US National Center for Healthcare Statistics. The patients/participants provided their written informed consent to participate in this study.

## Author contributions

XS, LZ, and CG: conceptualization. HZ and LS: statistical tests, data curation, and software. HZ, LS, and LZ: methodology. JL and YL: visualization. HZ, LZ, JL, YL, ZC, and SW: writing—original draft. HZ, XS, and CG: writing—review and editing. XS and CG: supervision and project administration. All authors contributed to the manuscript and approved the version submitted for publication.
