# Supplementary material for: The role of periodontitis in the link between alpha-tocopherol intake and cognitive performance: A mediation analysis in older adults
Source: Front Aging Neurosci. 2023 Mar 9;15:1129095. doi: 10.3389/fnagi.2023.1129095 (PMC10034200; doi:10.3389/fnagi.2023.1129095)
Supplement: Supplementary file 1 [file Table_1.DOCX]

Supplementary Material

The role of periodontitis in the link between alpha-tocopherol intake and cognitive performance: a mediation analysis in older adults

Heming Zhang^1, 2†^, Li Sun^1†^, Lin Zhang^3^, Jiangjing Li^1^, Yongfei Liu^1^, Zhiyang Chen^1,2^, Shuang Wang^1^, Changjun Gao^1*^, Xude Sun^1*^

*** Correspondence:** Xude Sun: sunxudes@163.com, Changjun Gao: gaocj74@163.com

# Supplementary Figures


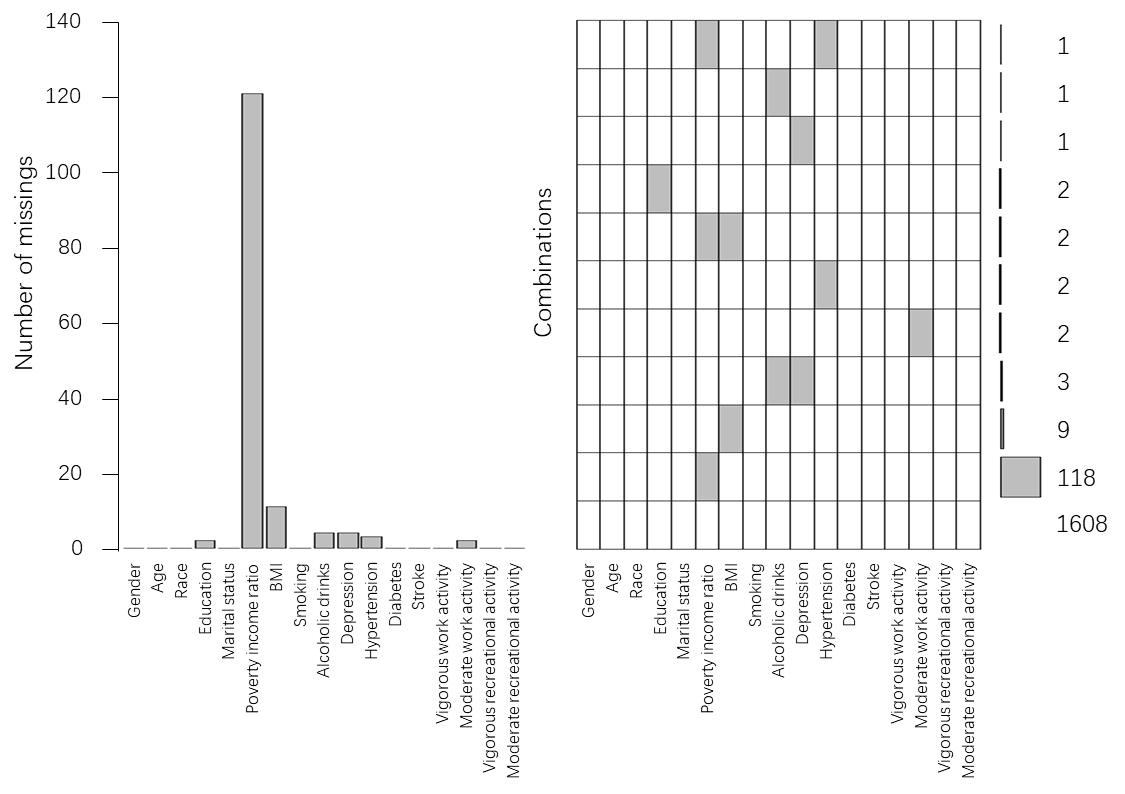


**Supplementary Figure S1.** Missing data of included participants.
